# Abstract of the Proceedings of the Buffalo Medical Association

**Published:** 1863-10

**Authors:** J. F. Miner

**Affiliations:** Secretary


ART. III.—*Abstract of the Proceedings of the Buffalo Medical Association.*

TUESDAY EVENING, September 1st, 1863.

Dr. Congar, President, in the Chair. Reading of the minutes of the last meeting postponed.

*Dr. Gay* remarked that he would attempt to entertain the Association for a few minutes, if no one else had anything special to present, with the subject of Fractures.

Fracture of the lower end of the radius *(colles fracture)* was quite common, but he had recently seen one complicated with dislocation of the ulna. Had looked through "Hamilton on Fractures and Dislocations," and found that such accident is not recognized. Thought possibly this might be due to accident rather than design. Would not speak at length of the treatment adopted, as it would not interest the Association. Used a modification of Hamilton's splint, and thought there could be no better.

Would inquire if any member of the Association had used Malgaign's apparatus, consisting of a ring with a pointed screw which is made to penetrate to the bone, and thus insure complete apposition of the displaced fragments. Thought it might be useful in cases of fracture of the tibia and fibula, with displacement of the upper fragment of tibia.

Also remarked that fracture of the neck of the thigh bone in young persons was regarded as a very rare accident. He had recently seen one such case.

Has had some experience in the treatment of fracture of the clavicle. Generally uses adhesive plaster. Has observed in the treatment of fractures generally that the position is best in which is the least pain, and proposes to dispense with constraining retentive apparatus; bones would gradually assume proper position and unite with good results.

*Dr. Cronyn* remarked that possibly a do-nothing plan of treatment would do well in some cases, but thought Dr. Gay would do well to consider the differences in the different points of fracture of the clavicle, otherwise he would have deformity and possibly suit for mal-practice. Related a case where the figure of eight bandage was carefully applied, and though the patient was very restless and impatient, yet the recovery was complete and without deformity. Spoke also of the different plans of treatment applied to fractures, and thought that no one, could be made applicable to all cases.

*Dr White* said, that fearing silence on the part of the members might be construed into endorsement by the Association of the views expressed upon the subject of fractures, he would make a brief reply, though he was sorry that some one more engaged in this department than himself should not be willing to conduct the discussion. In our efforts to improve we must be careful that we do not revolutionize. It appeared conclusively settled, that in oblique fractures some sort of retentive apparatus was not only conducive to comfort, but actually necessary to insure favorable result. He would not be very strenuous as to the kind, but that broken bones should be supported by some sort of retentive dressing, there could be no doubt. Young practitioners should not be deceived with the statement that an easy position was a safe and proper one, and that broken bones would always assume, *unaided*, a proper and natural position. If displacement had been produced, a careful adjustment should be made, and efficient measures adopted for retention. It was not always necessary to make immediate adjustment; the provisional callus, as it is called, is formed gradually, and previous to the eighth day final adjustment may be made, considerable indulgence granted until that time; but bones which have been badly displaced have no power or tendency voluntarily to assume a position from which they have been violently removed and return prevented by inflammatory effusions or mechanical obstacles. If it is only intended to say that we have been instructed to depend too much upon apparatus, and have used it unnecessarily, objection will not be taken; but if Dr. Gay intends to say that we do not require any at all, then he is holding to extreme views and cannot be sustained by the experience of the most extensive and accurate observers.

Malgaigne's apparatus, to which Dr. Gay referred, so far as he knew, had never been adopted by any surgeons of this country, and Malgaigne himself on account of his extreme views and practices had been denominated by the profession of his own country, the Tiger of Surgery. To speak favorably of an apparatus so harsh and painful in its application, designed to compel coaptation of bones by the most powerful mechanical force, and in the next sentence propose to dispense will all retentive apparatus upon the ground that bones would gradually assume a natural position, voluntarily, and unite without deformity, is advocating the two extremes at the same time, both of which are manifestly untenable.

*Dr White* also remarked, that the case of vulvo-vesical fistula, reported at a previous meeting, the result of which was at that time uncertain, had terminated favorably, and the patient now sleeps the entire night or rides from Niagara Falls, her place of residence, to this city, retaining her urine. It would be recollected that a flexible catheter was used instead of the one recommended by Dr. Sims. It was fastened to the thigh by adhesive plaster, so as not to become displaced. Thought this would answer equally well in almost all cases.

*Dr. Jansen* related a case of a young girl, fourteen years old, whom he was called to visit with Dr. Felgemacher. Found pulse small, lips blue, and complaining of severe pain in the left breast. Prescribed whisky and morphine. Dr. F. thought it was fictitious altogether—was because she did not like to be confirmed in the church. Prescribed for cyanosis. The child soon died, and they did not know the cause of death. No *post-mortem* examination permitted.

*Dr. Gay* related a case where a boy fell, striking upon the belly, and was dead in twenty minutes. *Post-mortem* examination not allowed.

*Dr. Jansen* mentioned an instance of sudden death after being run against by a car. The body was not bruised. Death took place immediately. Upon *post-mortem* examination the liver was found divided completely.

*Dr. Lockwood* introduced the subject of diarrhœa and dysentery, and spoke of its general prevalence in severe form, and also cholera infantum. Related the particulars of one case of dysentery which proved fatal, and appeared wholly beyond control by medication. He gave two grains opium and half a grain of calomel every two hours, and at the same time anodyne injections; but opium seemed to exert no influence in suspending the discharges, or the mercury in changing their character. The dejections were slime, with but little blood. Later in the disease gave stimulants freely. One injection was composed of nit. silver grs. x; water ℥i. This produced considerable pain, and was attended by no benefit.

The disease had appeared with him in its more intractable form, and many severe cases had come under his care. He had made use of the more common and also of the uncommon remedies, and had seen his cases terminate favorably in almost all instances, yet had been constantly under the impression that the disease was generally assuming a very persistent type. Had introduced the subject for the purpose of learning the opinions of others concerning it, both of its nature and proper treatment.

*Dr. Wyckoff* related one case seen with Dr. Storck. After the first visit Dr. S. took charge of the patient. When first visited he had been having dysenteric stools for some hours; was in great pain, had chills, fever, tenesmus, and the ordinary symptoms of dysentery. After Dr. S. had been with him about half an hour he passed at once two quarts of brine water. Ordinary treatment was adopted, or what he calls ordinary—opium and calomel. The peculiarity of the case was the combination of symptoms.

*Dr. Rochester* said there was unquestionably a great deal of diarrhœa and dysentery, some cases mild, and requiring little treatment, and others severe, like the ones described. He had lost some patients, and found the disease very intractable. His treatment had been of various kinds, and he had grown skeptical as to the influence of medication. Thought there was not more successful treatment than the saline cathartic and opium plan, especially in cases of febrile action. The sulphuric acid treatment which he had tried both in private and hospital practice, signally failed. Some times he had seen the disease in malarial form, requiring quinine for its cure, which he gave with opium. He often prescribed grain doses of morphine with calomel, with good results. Opiate suppositories were often very useful. Saline cathartics with opium would usually control the disease, but intractable cases would occur to every practitioner. If he was to give preference to any one plan, it would be after saline cathartics to give opium and quinine.

He had also tried in hospital a plan of treatment recommended by Dr. O'Shannesey of India. It consisted in administering ipecac in full doses—in one or two dram doses, emises to be prevented by previous administration of a full dose of tinc. opium. The opium did not prevent vomiting in the cases he had tried. The reasons for not trying it in private practice are sufficiently obvious. It had also been said by Dr. Buck of New York that the disease had been cut short by this plan of treatment, who gave illustrative cases, one of which was, a woman who had been sick ten days.

After the adoption of this plan, convalescence commenced immediately. Had himself had but little experience in this, and had only mentioned it thinking others may have had more.

*Dr. Wyckoff* spoke favorably of the cathartic plan of treament in adults; thought more highly of it than of any other. Had never adopted it with children.

After a spirited discussion concerning the time of publishing the proceedings, *voted* to adjourn.

J. F. MINER, *Secretary.*

